# RETRACTED: Xu et al. Cr–Diamond/Cu Composites with High Thermal Conductivity Fabricated by Vacuum Hot Pressing. *Materials* 2024, *17*, 3711

**DOI:** 10.3390/ma17236013

**Published:** 2024-12-09

**Authors:** Qiang Xu, Xiaodie Cao, Yibo Liu, Yanjun Xu, Jiajun Wu

**Affiliations:** 1 Central Iron & Research Institute, Beijing 100081, China; xuqiang@gangyan-diamond.com (Q.X.); liuyibo@gangyan-diamond.com (Y.L.); 2 Beijing Gang Yan Diamond Products Company, Beijing 102200, China; 3 College of Engineering, Shantou University, Shantou 515063, China; 23xdcao@stu.edu.cn

The journal retracts the article titled “Cr–Diamond/Cu Composites with High Thermal Conductivity Fabricated by Vacuum Hot Pressing” [1], cited above.

Following publication, the authors contacted the Editorial Office to raise concerns relating to the unauthorized use of propriety data presented within this article [1].

Adhering to our complaints procedure, the Editorial Office and Editorial Board conducted an investigation that confirmed that appropriate permission to publish the underlying data was not obtained, and that this publication breached the embargo period as set out by Beijing Gang Yan Diamond Products Company. As retroactive permission could not be obtained, the Editorial Board and the authors have decided to retract this article as per MDPI’s retraction policy (https://www.mdpi.com/ethics#_bookmark30, accessed on 3 December 2024).

This retraction was approved by the Editor-in-Chief of the *Materials* journal.

The authors agreed to this retraction.
